# Functional Alignment Achieved a More Balanced Knee After Robotic Arm-Assisted Total Knee Arthroplasty than Modified Kinematic Alignment

**DOI:** 10.3390/jcm14030820

**Published:** 2025-01-26

**Authors:** Hong-Yeol Yang, Jong-Keun Seon, Ji-Hyeon Yim, Dong-Hyun Lee, Eun-Kyoo Song

**Affiliations:** 1 Department of Orthopaedic Surgery, Chonnam National University Medical School and Hospital, 322, Seoyang-ro, Hwasun, Chonnam 58128, Republic of Korea; stephano.h.yang@gmail.com (H.-Y.Y.); seonbell@chonnam.ac.kr (J.-K.S.); 2 Joint & Arthritis Research, Department of Orthopaedic Surgery, Segyero Hospital, 77, Jangsin-ro, Gwangsan-gu, Gwangju 62224, Republic of Korea; presid50@naver.com (J.-H.Y.); j2sd1206@hanmail.net (D.-H.L.)

**Keywords:** functional alignment, modified kinematic alignment, gap balance, clinical outcomes, total knee arthroplasty, robotic arm-assisted total knee arthroplasty

## Abstract

**Background**: The aim of this study was to evaluate the balance in extension and flexion achievable after total knee arthroplasty (TKA) using a modified kinematic alignment (KA) plan and the subsequent balance achievable after adjusting the component based on the functional alignment (FA) principle. **Methods**: This retrospective cohort study included 100 consecutive patients who underwent primary TKA for knee osteoarthritis through an image-based robotic system in a single center between October 2021 and February 2022. Whether modified KA or FA could achieve a balanced knee was evaluated by assessing the ligament balance in the medial and lateral compartments using a robotic system at extension and 90° flexion. Balance was defined as a difference of ≤2 mm between the compartments. Component positioning was adjusted within limits based on the functional positioning principles to achieve balance. Implant positioning and balance in extension and 90° flexion were compared between the modified KA plan (n = 100) and after FA adjustments (n = 100). **Results**: FA achieved significantly better balance in extension (FA, 99.0% vs. modified KA, 86.0%; *p* = 0.001) and flexion (98.0% vs. 43.0%; *p* < 0.001) than the modified KA plan. The mean difference in gap balance in extension (FA, 0.1 mm vs. modified KA, 0.6 mm; *p* = 0.001) and flexion (0.1 mm vs. 2.3 mm; *p* < 0.001) was also significant between the two techniques. The femoral component was positioned more externally rotated relative to the transepicondylar axis (FA, 2.5° vs. modified KA, 0.0°; *p* < 0.001) to obtain balanced targets. There were significant improvements in the patient-reported outcome measures between preoperative and postoperative assessments two years after TKA (all *p* < 0.05). **Conclusions**: FA consistently achieved superior balance in both extension and flexion following TKA compared with modified KA without altering the soft tissue envelope, leading to significant improvements in clinical outcomes at the two-year follow-up.

## 1. Introduction

Historically, efforts have been focused on restoring the neutral mechanical axis (MA) to achieve a reliable and successful total knee arthroplasty (TKA) [1,2]. However, due to variations in normal knee anatomy, this technique often alters the patient’s natural knee orientation in all three dimensions to align the joint line perpendicular to the MA [3]. While this approach has demonstrated good long-term outcomes and longevity, it can lead to significant levels of patient dissatisfaction, affecting up to 20% of otherwise uncomplicated cases of TKA [4,5,6,7].

In recent years, there has been a growing interest in a more personalized approach to TKA to restore the native knee anatomy to address patient dissatisfaction [8,9]. Kinematic alignment (KA) has been suggested as an alternative to MA alignment, with the goal of restoring the pre-arthritic limb and joint line alignment based on each patient’s individual anatomy [10,11,12,13]. Marked varus alignment of the tibial component should be avoided in kinematically aligned TKA due to the risk of early failure; however, some studies have shown no difference in implant survival or function between aligned and misaligned TKA [14,15,16,17]. Considering these uncertainties, Vendittol et al. proposed a modified technique for kinematic TKA that utilizes an algorithm to adjust for more extreme patient anatomy that could potentially jeopardize the long-term success of the implant [18]. Advancements in computer-assisted technologies have led to the development of hybrid alignment techniques that combine KA within a safe alignment range and well-balanced soft tissue. Some surgeons have adopted this approach and reported satisfactory clinical outcomes [14,19,20,21,22,23,24].

The principle of achieving a balanced knee has long been widely regarded as a cornerstone of successful TKA. There is increasing evidence suggesting that tibiofemoral compartmental balancing is associated with kinematics after TKA [25,26,27,28]. The drive toward the adoption of advanced robotic systems reflects an effort to enhance the precision of gap balancing and bone cutting in TKA [29]. These innovations leverage preoperative computed tomography (CT) scans and intraoperative kinematic assessments integrated with patient-specific alignment strategies to refine surgical outcomes [30,31]. Numerous studies have corroborated that robotic arm-assisted systems deliver superior accuracy and efficiency in gap balancing relative to traditional manual techniques [32,33,34]. Functional alignment (FA) is considered an evolution of the KA approach that is designed to restore the joint plane and obliquity and balance the knee flexion-extension gap by fine-tuning adjustments of the components in positions that minimally compromise the periarticular soft tissue envelope [35,36]. The principles of functional positioning are based on planning software that provides predicted tibiofemoral gap data, allowing for adjustments in the extension and flexion gaps across all three planes [37].

This study aimed to assess the balance achievable with a modified KA plan and to evaluate the improvements in balance obtained through adjustments based on functional positioning principles. We compared the balance determined on a virtual model using an image-based robotic-assisted system and examined the difference in balance between the two alignment techniques. Our hypothesis was that FA would result in superior knee balance in extension and a flexion gap following TKA compared with modified KA.

## 2. Materials and Methods

### 2.1. Patients

**Figure 1:**
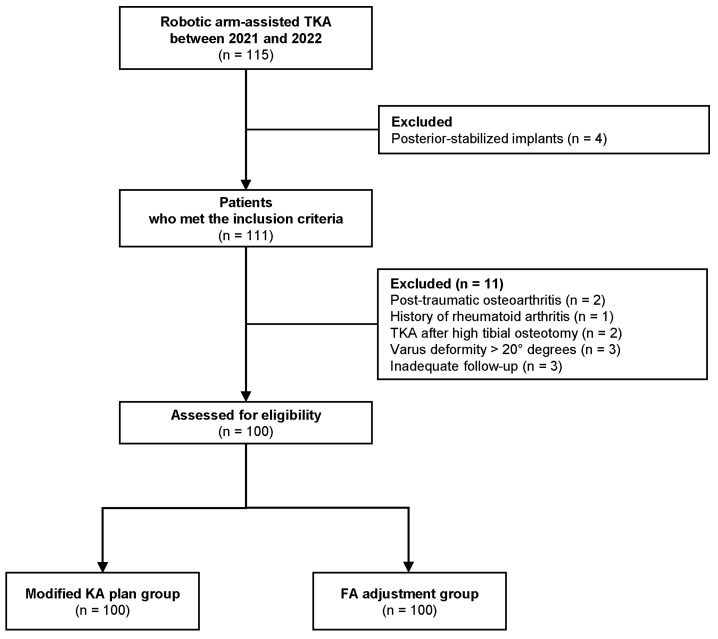
Flow chart describing the patient enrollment process in the study.

This study was approved by the institutional review board of our institution. We retrospectively reviewed 115 patients with end-stage knee osteoarthritis who underwent a primary robotic arm-assisted TKA (Stryker Triathlon, Mahwah, NJ, USA) using the Mako robotic system (Stryker, Kalamazoo, MI, USA) between October 2021 and February 2022. The functional positioning principles were applied after using a modified KA plan with the assistance of the robotic system. The inclusion criteria were as follows: patients who underwent robotic arm-assisted TKA for knee osteoarthritis using a cruciate-retaining (CR) prosthesis and a follow-up period of at least two years after operation. Patients were excluded if they had a varus deformity exceeding 20°, inflammatory arthritis, posttraumatic osteoarthritis, previous ipsilateral knee surgery, or incomplete follow-up data. The final cohort consisted of 100 patients in the complete analysis (Figure 1).

### 2.2. Balancing

**Figure 2:**
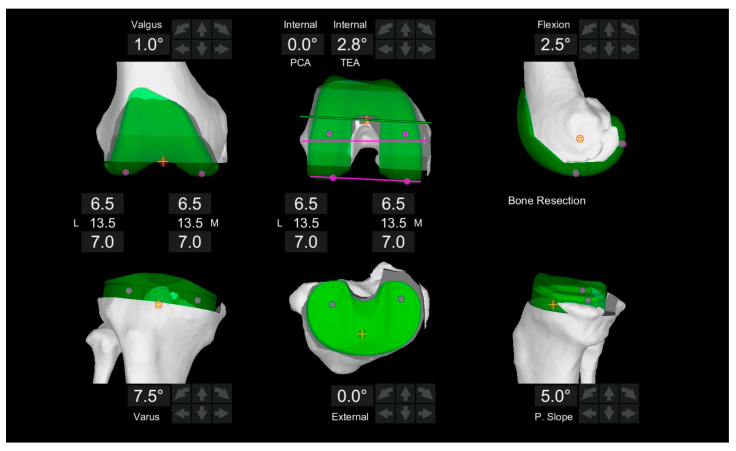
CT-based preoperative three-dimensional joint line orientation of the femur and tibia. Coronal alignment of the femoral and tibial components was planned by equalizing medial and lateral bone resections, aligning with the preoperative MPTA and LDFA. In this case, MPTA is 7.5° (varus), LDFA is −1.0° (valgus), and the posterior tibial slope (PTS) is 5.0°.

All operative procedures were conducted by a single experienced arthroplasty surgeon. Preoperative CT scans were analyzed with Mako software to create a 3D knee model for virtual implant placement guided by KA principles (Figure 2) [38].

**Figure 3:**
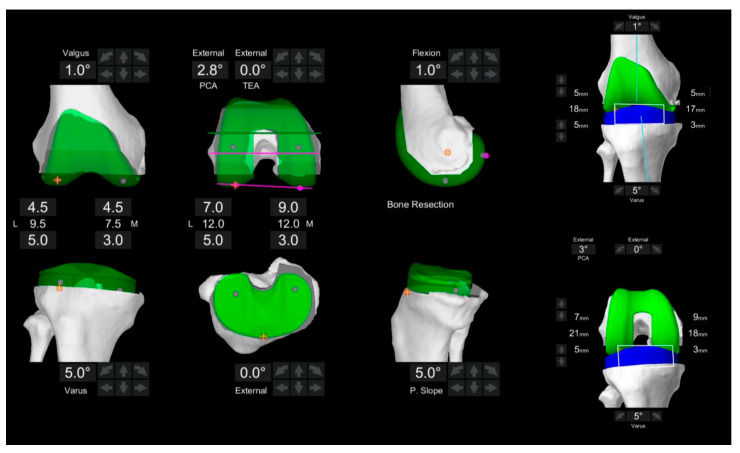
Intraoperative gap assessment in extension and flexion of a modified kinematic alignment plan that restricts the coronal osteotomy of the proximal tibia and distal femur to within ±5°. The tibial component alignment was set at 5.0° (varus) in a coronal alignment, while the femoral component coronal alignment was set at −1.0° (valgus) and 2.8° externally rotated relative to the posterior condylar axis (PCA). This indicates that the anatomic placement of the TKA will be tight medially in flexion and extension.

**Figure 4:**
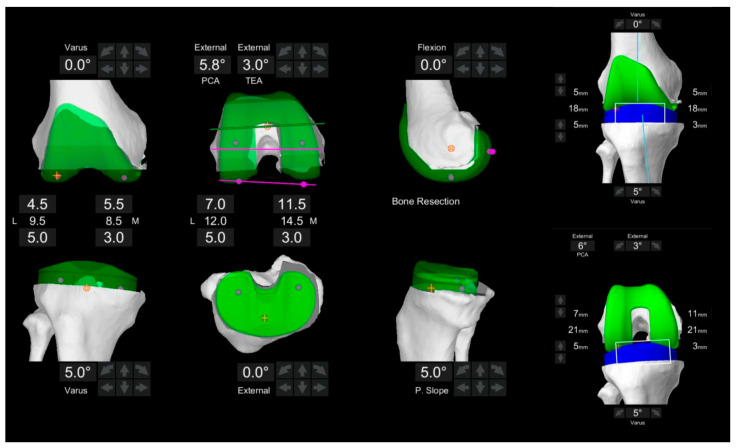
Intraoperative gaps after component adjustment in extension and flexion following functional alignment. The target gaps are shown on the right. The tibial component alignment was set at 5.0° (varus) in a coronal alignment, while the femoral component coronal alignment was set at 0° and 5.8° externally rotated relative to the posterior condylar axis (PCA). Due to soft tissue laxity, the femoral component was further externally rotated to achieve balance targets. Balanced extension and flexion gaps were successfully achieved in both compartments.

Balancing was adjusted during the surgery based on two different alignment techniques. Modified KA refers to resections that were adjusted according to the patient’s anatomy if the measured angles deviated beyond a predetermined “safe range” of either a combined coronal orientation within ±5 degrees of neutral and/or independent femoral or tibial cuts within ±5 degrees (Figure 3) [18]. Subsequently, functional positioning principles are applied, utilizing a tibia-first technique and gap balancing to align the femur relative to this reference (Figure 4). Soft tissue laxity was assessed intraoperatively, and virtual gaps were evaluated using the robotic platform to elucidate if the two alignment techniques achieved knee balance. Component positioning was adjusted accordingly. Gap balance was analyzed in extension (medial-lateral gap difference at 10° flexion) and flexion (medial-lateral gap difference at 90° flexion), and balance was defined as a difference of ≤2 mm. We aimed to achieve balanced extension gaps of 18 mm in the medial and lateral compartments with a flexion gap that was 2−3 mm greater than the extension gap. Bone cuts were performed following virtual balancing, and trial implants were inserted. Soft tissue balance was reassessed, and the final polyethylene bearing thickness was determined based on coronal and sagittal laxity.

### 2.3. Radiographic Evaluation

Standardized anteroposterior, lateral, and Merchant views of the knee and AP long-leg standing weight-bearing radiographs were obtained preoperatively and postoperatively. The radiographic evaluation included the assessment of the coronal alignment of the lower limb (mechanical hip-knee-ankle axis [mHKA] angle), the medial proximal tibial angle (MPTA), and the lateral distal femoral angle (LDFA). The mechanical HKA angle was measured as the angle formed by the mechanical axes of the femur and the tibia (positive for varus). The MPTA was defined as the medial angle between the mechanical axis of the tibia and the joint line of the proximal tibia, while the LDFA was defined as the lateral angle between the mechanical axis of the femur and the joint line of the distal femur.

### 2.4. CPAK Classification and CPAK Classification Matrix

Constitutional knee phenotypes were classified using the coronal plane alignment of the knee (CPAK) system described by MacDessi et al. [3]. The constitutional alignment, represented by the arithmetic HKA (aHKA) and joint line obliquity (JLO), was calculated using the following formulas: aHKA = MPTA − LDFA and JLO = MPTA + LDFA.

### 2.5. Clinical Evaluation

The clinical assessment was based on evaluations performed preoperatively and two years after the operation. Clinical outcomes were determined by patient-reported outcome measures (PROMs) based on the Knee injury and Osteoarthritis Outcome Score (KOOS) and Forgotten Joint Score (FJS). Furthermore, clinical evaluations of range of motion (ROM) were also performed using a goniometer at the same time points.

### 2.6. Statistical Analysis

Native coronal alignment phenotypes, which were classified using the CPAK system, were visualized with scatterplots to illustrate their distribution within the study cohort. Paired and independent *t*-tests were used for normally distributed variables, while the Wilcoxon signed-rank test and Mann-Whitney U test were employed for non-normally distributed data. The chi-square test or Fisher’s exact test was used to compare differences in categorical variables. The threshold for significance was set at *p* < 0.05.

## 3. Results

**Figure 5:**
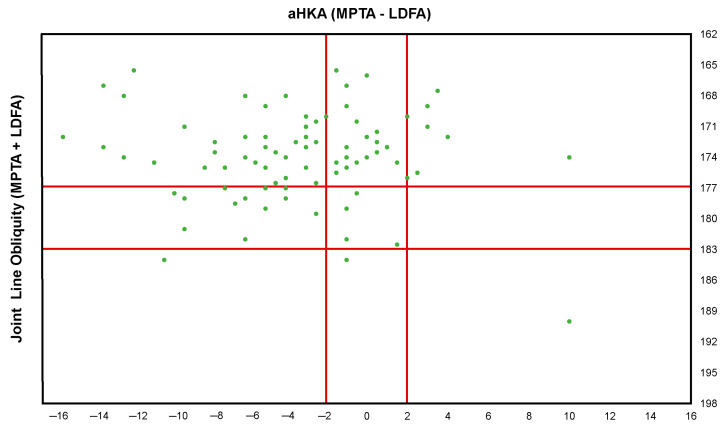
Distribution of native coronal plane alignment of the knee phenotype in the study cohort.

The demographic characteristics and clinical data of the patients are summarized in Table 1. Figure 5 illustrates the distribution of knee phenotypes classified according to the CPAK system. Type I was the most common preoperative phenotype, accounting for 47.0% (n = 47) of cases, characterized by constitutional varus alignment and apex distal JLO orientation.

FA achieved significantly better balance in extension (FA, 99.0% vs. modified KA, 86.0%; *p* = 0.001) and flexion (98.0% vs. 43.0%; *p* < 0.001) than modified KA (Table 2). The mean difference in gap balance in extension (FA, 0.1 mm vs. modified KA, 0.6 mm; *p* = 0.001) and flexion (0.1 mm vs. 2.3 mm; *p* < 0.001) was also significantly different between the two techniques. Balanced extension and flexion gap could not be obtained within the component alignment boundaries in two patients (2.0%) who had severe constitutional varus deformity, necessitating additional soft tissue release. The femoral component was positioned with greater external rotation relative to the surgical transepicondylar axis (TEA) (FA, 2.5° vs. modified KA, 0.0°; *p* < 0.001) to achieve balancing targets (Table 3).

The mean range of motion improved from 120.8 (SD 10.1) to 130.1 (SD 9.0) (*p* < 0.001) two years after FA TKA. All KOOS scores were significantly improved (*p* < 0.001); pain from 42.0 (SD 10.7) to 84.5 (SD 9.0), symptoms from 48.1 (SD 11.8) to 85.1 (SD 8.5), daily activities from 41.1 (SD 10.1) to 79.1 (SD 8.8), sport and recreation from 23.1 (SD 5.4) to 25.9 (SD 9.1), and quality of life from 21.8 (SD 8.8) to 68.4 (SD 10.8). The postoperative FJS was 81.8 (SD 7.5), and no complications were reported within the first two years following the surgery.

## 4. Discussion

The principal findings of the present study were that FA achieved superior knee balance in both extension and flexion after TKA compared to modified KA, without altering the soft tissue envelope, and led to a marked improvement in clinical outcomes at two years postoperatively. Modified KA was unable to reliably balance both spaces compared with FA, and adjusting for soft tissue laxity often led to a more externally rotated femoral component to achieve balance.

The importance of achieving knee balance for successful TKA has been widely recognized [39]. Sappey-Marinier et al. have shown that KA matches the constitutional alignment of the knee for the extension gap using an arithmetic method, resulting in consistent balancing. However, varus morphotype knees show a substantial variation in gap widths at different joint positions [40]. Shatrov et al. have demonstrated that, in over half of the cases, KA was unable to achieve balanced TKA for varus knee osteoarthritis, particularly in the flexion gap [41]. In the present study, modified KA failed to achieve a balanced extension space in 14% of cases and a balanced flexion space in 57% of cases, consistent with the findings of previous studies [41,42,43]. Based on these findings along with the variability in soft tissue laxity profiles among individuals, a more personalized approach to TKA alignment that adapts to these variations in the soft tissue envelope is necessary.

FA is indeed considered an evolution of the KA method that is designed to restore the joint plane and obliquity and balance the knee flexion-extension gap with minimal compromise to the periarticular soft tissue envelope [35,38]. In the current study, with the FA technique, extension balance was achieved in 99% of cases, and flexion balance was achieved in 98% of cases without any modifications to the periarticular soft tissue envelope. The femoral component was positioned with greater external rotation to the TEA and greater flexion following FA adjustments than the findings of a previous study by Shatrov et al. These differences may partly be attributed to the use of a posterior-stabilized (PS) implant system in their simulation study. The complete release of the posterior cruciate ligament (PCL) increases the flexion gap by 4–5 mm, which could lead to a mismatch between the medial and lateral compartments if not adjusted for [44]. When using a CR prosthesis, as in our study—particularly with robotic arm-assisted TKA and a lower incidence of PCL injury—a tight medial flexion gap is more likely to be encountered. This study demonstrated that using virtual knee balancing and intraoperative soft tissue envelope data allows most TKAs to achieve balance without requiring soft tissue release. Functional positioning principles are anticipated to maximize the advantages of robotic arm-assisted TKA and increase the reliability of achieving a balanced knee. Improved balance has also been associated with better PROMs [42,45].

In the current study, FA consistently provided a balanced TKA using an image-based robotic-assisted system before performing soft tissue release, and the effect of achieving balance on improved clinical outcomes at two years postoperatively was demonstrated. Walkelin et al., in their multicenter study, have identified gap thresholds that provided clinically relevant and achievable targets for optimizing soft tissue balance in TKA, which were similar to the thresholds in our study, and demonstrated an association between balanced gaps and improved clinical outcomes [45]. Clark et al. have reported that using an FA technique that results in more anatomical component positioning in TKA leads to superior clinical outcomes, particularly in constitutional varus knees [36]. Given the majority of patients in the present study have CPAK type I, we believe it is crucial to utilize a more personalized TKA based on a robotic-assisted system. In fact, a wide range of gaps were generated using the modified KA approach, with a pronounced discrepancy between medial and lateral gaps compared to the FA principle. Our results align with two recent studies indicating that while laxity in the extension space could be predicted, the variability in the flexion space was considerably greater, necessitating a personalized approach. In this study, FA cases showed greater accuracy and precision in achieving postoperative alignment than the intraoperative plan, consistent with the findings of previous literature [30,46,47,48]. Furthermore, the improved functional outcomes were sustained for up to two years postoperatively. Recent systematic reviews have demonstrated significantly higher accuracy and improved clinical outcomes associated with robotic arm-assisted TKAs [49]. While long-term survivorship data for robotic arm-assisted TKA remains limited, registry data suggest improved early survival rates irrespective of surgeon experience or procedural volume [50].

Several limitations should be outlined in this study. First, it was a non-randomized, retrospective study based on the database of a single institute. Second, the follow-up period was not long enough to discuss long-term survivorship; therefore, a long-term prospective study with correlated clinical results is needed to substantiate our findings. Third, debate continues regarding the optimal targets for gap measurements, highlighting the need for further investigation and discussion of the gaps observed in this study. Fourth, this study did not reveal an effect of differential technique (modified KA or FA) on clinical outcomes. Nevertheless, the primary objective was to evaluate the consistency of each technique in achieving consistent balancing targets and to determine the differences in balanced gaps between the two techniques.

## 5. Conclusions

FA consistently achieved superior balance in both extension and flexion following TKA compared with modified KA without altering the soft tissue envelope, leading to significant improvements in clinical outcomes at the two-year follow-up. Our findings suggest that a more personalized approach is necessary for achieving consistent balance in TKA and improving functional outcomes after TKA. Future studies will be needed to assess the impact of this approach on implant survivorship.

## Figures and Tables

**Table 1:** Patient Demographic and Radiographic Characteristics *.

	Value
Age (yr)	69.6 ± 6.3
Female sex (number of patients)	76 (76.0)
Body mass index (kg/m^2^)	26.3 ± 4.1
ASA grade (number of patients)	
II	59 (59.0)
III	41 (41.0)
Side of operation (number of patients)	
Right	46 (46.0)
Left	54 (54.0)
Preoperative radiographic parameters	
mHKA † (deg)	9.5 ± 5.4
MPTA (deg)	85.1 ± 3.3
LDFA (deg)	88.6 ± 3.0
aHKA (deg)	−4.3 ± 2.2
JLO (deg)	173.7 ± 4.2
CPAK classification distribution (number of patients)	
Type I	47 (47.0)
Type II	29 (29.0)
Type III	5 (5.0)
Type IV	12 (12.0)
Type V	4 (4.0)
Type VI	0 (0)
Type VII	1 (1.0)
Type VIII	1 (1.0)
Type IX	1 (1.0)

* Values are presented as the mean ± standard deviation or number (%). † A positive angle represents varus alignment, and a negative angle represents valgus alignment. ASA, American Society of Anesthesiologists; mHKA, mechanical hip-knee-ankle angle; MPTA, medial proximal tibial angle; LDFA, lateral distal femoral angle; aHKA, arithmetic hip-knee-ankle angle; JLO, joint line obliquity; CPAK, Coronal Plane Alignment of the Knee.

**Table 2:** Intraoperative Gap Measures and Number of Knees Balanced in Modified Kinematic Alignment and Functional Alignment *.

	Modified KA	FA	*p* Value †
Gap Measures			
Medial extension (mm)	17.7 ± 2.0	18.8 ± 1.1	**<0.001**
Lateral extension (mm)	18.3 ± 1.7	18.8 ± 1.1	**0.008**
Medial flexion (mm)	19.4 ± 2.9	21.3 ± 0.8	**<0.001**
Lateral flexion (mm)	21.8 ± 2.2	21.5 ± 0.9	0.323
Medial−lateral difference in extension (mm)	0.6 ± 1.7	0.1 ± 0.3	**0.001**
Medial−lateral difference in flexion (mm)	2.3 ± 2.7	0.1 ± 0.5	**<0.001**
Number of Balanced Knees ‡			
Extension balance ≤ 2 mm	86 (86.0)	99 (99.0)	**0.001**
Flexion balance ≤ 2 mm	43 (43.0)	98 (98.0)	**<0.001**

* Values are presented as the mean ± standard deviation or number (%). † An independent *t*-test was used to analyze differences in radiological parameters. Bold indicates a significant difference. ‡ Assessment of gap balance in extension (difference between medial and lateral gaps at 10° flexion) and flexion (difference between medial and lateral gaps at 90° flexion) was performed. A gap balance within 2 mm was considered acceptable for balanced knee alignment. KA, kinematic alignment; FA, functional alignment.

**Table 3:** Intraoperative Alignment Observed Utilizing Modified Kinematic Alignment and Functional Alignment *.

	Modified KA	FA	*p* Value †
Femoral coronal alignment ‡ (deg)	−1.1 ± 2.4°	−0.9 ± 1.9°	0.310
Femoral sagittal alignment (deg)	1.5 ± 1.6°	1.8 ± 1.8°	0.155
Femoral rotation to TEA § (deg)	0°	2.5 ± 2.4°	**<0.001**
Tibial coronal alignment ‡ (deg)	3.9 ± 1.4°	4.2 ± 1.6°	0.145
Tibial slope (deg)	6.0 ± 0.9°	6.1 ± 1.1°	0.349

* Values are presented as the mean ± standard deviation. † An independent *t*-test was used to analyze differences in radiological parameters. Bold indicates a significant difference. ‡ A positive angle represents varus alignment, and a negative angle represents valgus alignment. § A positive angle represents external rotation, and a negative angle represents internal rotation. KA, kinematic alignment; FA, functional alignment; TEA, trans-epicondylar axis.

## Data Availability

The data presented in this study are available on request from the corresponding author. The data are not publicly available.
